# Predictive performance of triglyceride glucose index (TyG index) to identify glucose status conversion: a 5-year longitudinal cohort study in Chinese pre-diabetes people

**DOI:** 10.1186/s12967-023-04402-1

**Published:** 2023-09-15

**Authors:** Xiaojie Chen, Danfeng Liu, Weiting He, Haofei Hu, Wenjian Wang

**Affiliations:** 1 The Second School of Clinical Medicine, Southern Medical University, Guangzhou, China; 2 Department of Nephrology, Guangdong Provincial People’s Hospital, Guangdong Academy of Medical Sciences, Southern Medical University, 106 Zhongshan Er Road, Main Building, Room 1436, Guangzhou, 510080 Guangdong China; 3 Guangdong Academy of Medical Sciences, Guangzhou, China; 4 Department of Nephrology, The First Affiliated Hospital of Shenzhen University, Shenzhen, China; 5 Department of Nephrology, Shenzhen Second People’s Hospital, No.3002 Sungang Road, Futian District, Shenzhen, 518000 Guangdong China; 6 South China University of Technology, Guangzhou, China

**Keywords:** Triglyceride glucose index, Glucose status conversion, Prediabetes, Cohort study, Non-linear, Cox proportional-hazards regression

## Abstract

**Objective:**

Triglyceride glucose index (TyG index) has been recommended as an alternative indicator of insulin resistance. However, the association between TyG and regression from prediabetes to normoglycemia remains to be elucidated.

**Methods:**

This retrospective cohort study involved 25,248 subjects with prediabetes at baseline conducted from 2010 to 2016. A Cox proportional hazard regression model was designed to evaluate the role of TyG in identifying people at converting from prediabetes to normoglycemia. Cox proportional hazards regression with cubic spline functions and smooth curve fitting was used to dig out the nonlinear relationship between them. Detailed evaluations for TyG were also performed using sensitivity and subgroup analyse.

**Results:**

Among the included prediabetes subjects (n = 25,248), the mean age was 49.27 ± 13.84 years old, and 16,701 (66.15%) were male. The mean TyG was 8.83 ± 0.60. The median follow-up time was 2.96 ± 0.90 years. 11,499 (45.54%) individuals had a final diagnosis of normoglycemia.

After adjusting for covariates, TyG was negatively affecting the results of glucose status conversion in prediabetes people (HR 0.895, 95% CI 0.863, 0.928). There was a nonlinear connection between TyG and normoglycemia in prediabetes people, and the inflection point was 8.88. The effect sizes (HR) on the left and right sides of the inflection point were 0.99 (0.93, 1.05) and 0.79 (0.74, 0.85), respectively. Sensitivity analysis confirmed the robustness of these results. Subgroup analysis showed that TyG was more strongly associated with incident glucose status conversion in male, BMI ≥ 25. In contrast, there was a weaker relationship in those with female, BMI < 25.

**Conclusion:**

Based on sample of subjects evaluated between 2010 and 2016, TyG index appears to be a promising marker for predicting normoglycemic conversion among prediabetes people in China. This study demonstrates a negative and non-linear association between TyG and glucose status conversion from prediabetes to normoglycemia. TyG is strongly related to glucose status conversion when TyG is above 8.88. From a therapeutic point of view, it is meaningful to maintain TyG levels within the inflection point to 8.88.

**Supplementary Information:**

The online version contains supplementary material available at 10.1186/s12967-023-04402-1.

## Background

Diabetes is one of the most serious and common chronic diseases worldwide, which causes life threatening and disabling complications, and contributes to health burden especially in a developing country like China. It is deduced that 537 million people come down with diabetes in 2021, and this number will reach 643 million at the end of 2030, and 783 million at the end of 2045. In addition, 541 million people are deduced to have impaired glucose tolerance in 2021. It is also deduced that more than 6.7 million people aged 20–79 will die from diabetes-related causes in 2021 [1]. While prediabetes is a high-risk state for diabetes development. Prediabetes comprise two characteristics of impaired fasting glucose (IFG) and/or impaired glucose tolerance. The definition of IFG according to World Health Organization (WHO) using fasting plasma glucose (FPG) is in the range of 6.1–6.9 mmol/L, while according to the American Diabetes Association (ADA) FPG is in the range of 5.6–6.9 mmol/L with a lower threshold [2, 3]. Two recent studies have revealed that the prevalence of prediabetes is 35.2–35.7% [4, 5] in Chinese population with the ADA standard, which is greater than that with the WHO standard. Without any health intervention, approximately 30% of people with prediabetes will experience diabetes within 5 years [6]. Compared to those with normoglycemic people, even the progression of diabetes is prevented or delayed, both microvascular and macrovascular disease appears to be more prevalent in people with prediabetes [7]. Therefore, it is convincing that true prevention of diabetes and its complications is put down to the reversal of prediabetes and the restoration of normoglycemic regulation.

In a recent systematic review [8] which included 47 studies on prediabetes reversion, showed that glucose-regression rates in prediabetes were between 33 and 59% at 1–5 years of follow-up, and between 17 and 42% at 6–11 years of follow-up. In another study it showed that reversion rates ranged from 21 to 68% (Intervention and control/placebo arms) over a median follow up period of 2.7 years with or without interventions which includes medications, dietary or physical activity/exercise improvements, dietary supplements (vitamin D, magnesium, and Larginine), and Chinese medicine[9]. Recent literatures showed that female, as well as those with higher education were more likely to have normoglycemia reversion. While people with BMI ≥ 25 kg/m^2^, abdominal obesity, heavy drinking, hypertension, dyslipidemia, a family history of diabetes, higher baseline FPG, and higher baseline HbA1c lowered the probability of normoglycemia reversion [10–12]. Lifestyle modification and medications were also considered to be the modifiable factors of normoglycemia reversion with prediabetes status [13, 14].

Insulin resistance (IR) is a key feature of prediabetes and a precursor to the progression of type 2 diabetes mellitus (T2DM) [15], so a simple and effective method to assess IR status is important for implementing prevention and management strategies. The euglycemic-hyperinsulinemic clamp (EHC) has become the gold standard for direct assessing and identifying β cell sensitivity to insulin response. However, the EHC is invasive, time-consuming, expensive, and requires frequent blood drawing. The homeostatic model assessment for IR (HOMA-IR) and some other fasting insulin-based indexes are also inappropriate for screening IR in most health facilities because of their time-consuming nature, cost and requirement of skilled manpower. So indexes using routine physical examination indicators instead of insulin-based indicators have been supposed to be superior as alternatives for insulin measurements. Recently, triglyceride glucose index (TyG Index) has been identified as an novel and early indicator of IR [16] and can be easily applied in health facilities. Evidence showed that progression from prediabetes to diabetes was associated with severe deterioration of β-cell dysfunction. Improving insulin sensitivity and β-cell function has revealed to be beneficial for prediabetes stabilization and reversion from prediabetes to normoglycemia [17]. For TyG index, it is a compound index of fasting blood glucose and triglyceride, as a surrogate estimate of insulin sensitivity and β-cell function that could be applied constantly in large-scale observational and/or interventional cohorts [18], we hypothesized that TyG might also predict the reversion to normoglycemia from prediabetes. However, by consulting previous literature, the link between TyG and reversion to normoglycemia has not yet been widely explored in the prediabetes population.

To our best knowledge, there was only one study [19] has been conducted to assess the association between TyG and glucose status conversion among reproductive-aged women in Indonesia with 5 years of follow-up time and it only included reproductive-aged women in prediabetes state, which maybe limited when apply to the Chinese prediabetes population. Simultaneously, the effect of the intervention on TyG on the prevalence of glucose status conversion from prediabetes to normoglycemia also needs to be further explored. Hence we conducted a cohort study to investigate whether the TyG is independently associated with glucose status conversion in Chinese prediabetes people.

## Materials and methods

### Study design

This was a retrospective cohort study using records from a computerized database established by Chinese researchers (Chen et al.). The target independent variable was TyG at baseline. The outcome variable was normoglycemia (dichotomous variable: 0 = non-normoglycemia (pre-diabetes or diabetes or self-report diabetes), 1 = normoglycemia).

### Data source

The raw data was downloaded freely from the DATADRYAD database (www.datadryad.org) provided by Chen et al. [20]. Data was from a published article: Association of body mass index and age with incident diabetes in Chinese adults: a population-based cohort study. This is an open-access article which is given in accordance with the Creative Commons Attribution Non-Commercial (CC BY-NC 4.0) license and enables people to share, remix, modify, and create a derivative work from this work for non-commercial purposes as long as the author and source are credited [20].

### Study population

The source data were extracted from a computerised database established by the Rich Healthcare Group in China, which includes all medical records for participants who received a health check from 2010 to 2016. The present analysis included all study participants ≥ 20 years old and ≥ 2 visits between 2010 and 2016 (n = 685277). Exclusion criteria was as follows: (1) No available weight and height measurements (n = 103946), No available information on gender (n = 1), extreme BMI values (< 15 kg/m^2^ or > 55 kg/m^2^) (n = 152), or no available fasting plasma glucose value (n = 31370) at baseline; (2) Participants diagnosed with diabetes at baseline (2997 participants diagnosed by self-report, 4115 diagnosed by a FPG ≥ 7.0 mmol/L); (3)With visit intervals < 2 years (n = 324233); (4)With undefined diabetes status at follow-up (n = 6630). At last, the original study included a total of 211833 participants.

**Fig. 1:**
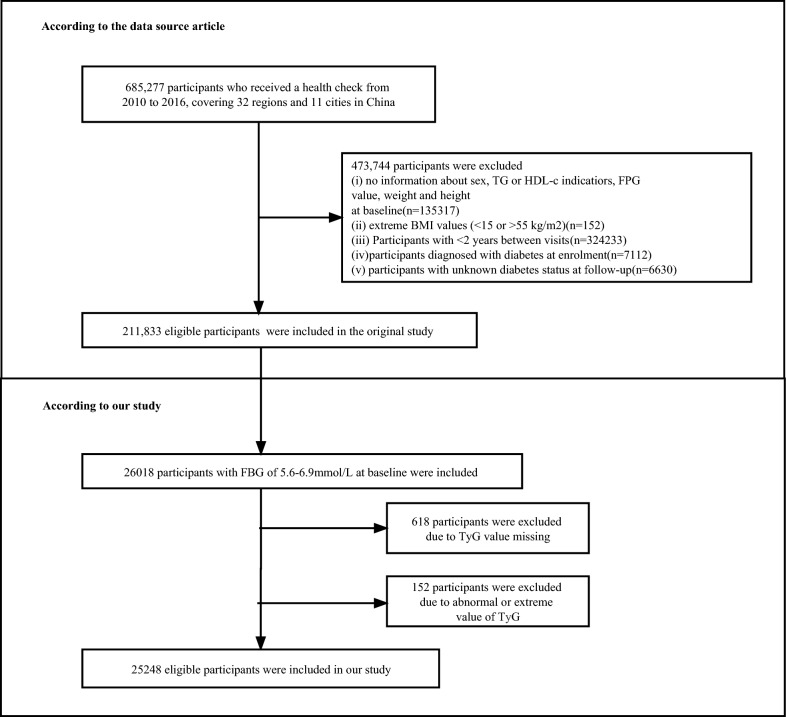
Study design and flowchat of study participants

A total of 26,018 participants with baseline FPG of 5.6–6.9 mmol/l were enrolled in our study. Pre-diabetes is defined as an FPG level of 5.6–6.9 mmol/L according to the ADA criteria. After excluding participants due to TyG value missing (n = 618) and participants due to abnormal or extreme value of TyG (n = 152), In the end, 25248 eligible participants were left for data analysis (see flowchart for details in Fig. 1).

The original study was initially approved by the Rich Healthcare Group Review Board. The data was retrieved retroactively and the institutional ethics committee did not ask for informed consent or approval for the retrospective study.

### Variables

#### Triglyceride glucose index (TyG index)

TyG Index is calculated by the following formula [21]. TyG Index = Ln (fasting glucose (mg/dL) × triglycerides (mg/dL)/2).

#### Outcome measures

The interesting outcome variable in our investigation was glucose status conversion from prediabetes to normoglycemia (dichotomous variable: 0 = non-normoglycemia, 1 = normoglycemia). FPG < 5.6 mmol/L without self-reported diabetes was defined as incident normoglycemia.

#### Covariates

We select the covariates according to our clinical experience and the previous literature. The covariates were as follows: (1) continuous variables: age, body mass index(BMI), systolic blood pressure (SBP), diastolic blood pressure (DBP), total cholesterol (TC), high-density lipoprotein cholesterol (HDL-C), low-density lipid cholesterol (LDL-C), alanine aminotransferase (ALT), aspartate aminotransferase (AST), blood urea nitrogen(BUN), serum creatinine (Scr), fasting plasma glucose (FPG). (2) categorical variables: sex, smoking status, drinking status, family history of diabetes.

Information on demographic characteristics (age, gender), living habit (cigarette smoking and alcohol consumption levels), medical history and family history of chronic disease diseases were collected through the detailed questionnaire in each visit to the health check center. Height, weight and blood pressure were measured by trained staff. BMI was calculated as weight in kilograms divided by height in meters square (kg/m^2^). Standard mercury sphygmomanometers were applied to measure blood pressure. Total cholesterol (TC), serum triglyceride (TG), low-density lipoprotein cholesterol (LDL-C) and high-density lipoprotein cholesterol (HDL-C) were measured on an autoanalyzer (Beckman 5800). Fasting venous blood samples were collected after at least a 10 h fast at each visit. Plasma glucose levels were measured by the glucose oxidase method on an autoanalyzer (Beckman 5800).

### Statistical analysis

Participants were stratified by TyG quartiles. Quantitative variables with normal distribution were expressed as the mean ± standard deviation, and variables with skewed distribution were expressed as the median (quartile), and categorical variables were expressed as the frequency (percentage). We used the One-Way ANOVA test (normal distribution), the χ^2^ (categorical variables), or the Kruskal–Wallis H test (skewed distribution) to test for differences among different TyG quartile groups.

To assess the impact of modifiable risk factors in blood glucose changes, we used univariate and multivariate Cox proportional risk regression models to construct three models, including unadjusted model (crude model: unadjusted covariate) and minimum adjusted model (Model I: only adjusted sociodemographic variables, Including age, sex, BMI, systolic blood pressure, diastolic blood pressure) and fully adjusted models (Model II: adjusted covariates including age, sex, BMI, systolic blood pressure, diastolic blood pressure, ALT, AST, BUN, LDL-C, HDL-c, Scr, family history of diabetes, drinking status, and smoking status).

Moreover, sensitivity analysis was conducted to test the robustness of our results by deleting participants with BMI ≥ 25 kg/m^2^, or deleting participants who had consumed alcohol, or deleting paticipants who had smoked, or deleting participants with a family history of diabetes, in order to evaluate the quality and consistency of the results. Besides, a generalized additive model (GAM) was used to insert the continuity covariate into the equation (model III) in curvy form to ensure the robustness of the results.

In order to investigate the nonlinear relationship between TyG and glucose state transition, Cox proportional hazards regression model with cubic spline functions and the smooth curve fitting was used to state the non-linear relationship. Besides, the two-piecewise Cox proportional-hazards regression model was used to further elaborate the non-linearity between TyG and glucose status conversion.

The subgroup analyses were conducted using a stratified Cox proportional-hazards regression model across various subgroups (sex, age, SBP, DBP, BMI). Continuous variables as age (< 30, ≥ 30 to < 40, ≥ 40 to < 50, ≥ 50 to < 60, ≥ 60 to < 70, ≥ 70 years), BMI (< 25, ≥ 25 kg/m^2^), SBP (< 140, ≥ 140 mmHg), DBP (< 90, ≥ 90 mmHg) [22–24] were transformed to a categorical variable based on the clinical cut point. Then we adjusted each stratification for all factors (Models were adjusted for age, sex, BMI, AST, ALT, SBP, DBP, HDL-c, LDL-C, BUN, Scr, smoking status, drinking status, and family history of diabetes, but not adjusted for stratification variables in each model). At last, tests for interaction were conducted with and without interaction terms.

The number of participants with missing data of BUN, Scr, SBP, DBP, HDL-C, LDL-C, AST, ALT, Drinking status and Smoking status were 7 (0.03%), 7 (0.03%), 9907 (39.24%), 9280 (36.76%), 210 (0.83%), 14098 (55.84%), 2435 (9.64%), 1119 (4.43%), 16710 (66.22%), 16710 (66.22%), respectively. Missing data of covariants was handled by multiple imputations method. The imputation model included age, sex, BMI, SBP, TC, HDL-C, LDL-C, ALT, AST, BUN, Scr, Drinking status, Smoking status, Family history of diabetes. Missing data analysis procedures use missing-at-random (MAR) assumptions.

All results were written according to the STROBE statement [25]. Both R statistical software packages (http://www.r-project.org, The R Foundation), SPSS 27.0 (SPSS Inc., Chicago, IL, USA) and Empower Stats (X&Y Solutions, Inc., Boston, MA, http://www.empowerstats.com) were used to conduct all analyses. Statistical significance was defined as P-values less than 0.05 (two-sided).

## Results

### Baseline characteristics of participants

The baseline characteristics of study participants were listed in Table 1. The mean age was 49.27 ± 13.84 years old, and 16,701 (66.15%) were male. The mean TyG was 8.83 ± 0.60. During a median follow-up time of 2.96 ± 0.90 years, 11,499 (45.54%) individuals had experienced a final diagnosis of normoglycemia. Participants were devided into subgroups according to TyG quartiles (< 8.41, ≥ 8.41 to < 8.81, ≥ 8.81 to < 9.22, ≥ 9.22). Compared with the Q1 (< 8.41) group, age, BMI, SBP, DBP, Cholosterol, LDL-C, ALT, AST, BUN, Scr increased significantly in the Q4 (≥ 9.22) group, while HDL-C decreased in the Q4 (≥ 9.22) group. Besides, Q4 (≥ 9.22) group had a higher proportion of male, current and ever smokers, current and ever drinkers when compared to Q1 (< 8.41) group.

**Table 1:** The baseline characteristics of participants

TyG quartile	Q1 (< 8.42)	Q2 (8.42–8.81)	Q3 (8.81–9.22)	Q4 (> 9.22)	P-value
Participants	6312	6306	6317	6313	
Age (years)	45.37 ± 13.92	49.72 ± 14.33	50.95 ± 13.52	51.04 ± 12.77	< 0.001
BMI (kg/m^2^)	22.95 ± 3.03	24.49 ± 3.15	25.50 ± 3.22	26.28 ± 3.09	< 0.001
SBP (mmHg)	122.20 ± 16.66	126.58 ± 17.34	128.95 ± 17.56	131.09 ± 17.63	< 0.001
DBP (mmHg)	74.95 ± 10.57	77.57 ± 10.90	79.64 ± 11.01	81.35 ± 11.07	< 0.001
TC (mmol/L)	4.53 ± 0.83	4.87 ± 0.87	5.12 ± 0.91	5.35 ± 0.96	< 0.001
HDL-C (mmol/L)	1.41 ± 0.29	1.36 ± 0.31	1.29 ± 0.28	1.25 ± 0.29	< 0.001
LDL-C (mmol/L)	2.61 ± 0.64	2.87 ± 0.68	3.03 ± 0.71	3.04 ± 0.76	< 0.001
ALT (U/L)	16.30 (12.00–23.00)	20.10 (15.00–29.20)	24.10 (17.40–35.50)	29.50 (20.70–44.30)	< 0.001
AST (U/L)	23.23 ± 10.69	25.39 ± 10.40	27.54 ± 11.94	30.08 ± 13.79	< 0.001
BUN (mmol/L)	4.95 ± 1.28	4.99 ± 1.27	5.02 ± 1.22	5.00 ± 1.23	0.020
Scr (μmol/L)	69.19 ± 15.39	72.16 ± 15.92	73.81 ± 15.39	75.63 ± 16.51	< 0.001
Sex					< 0.001
Male	3335 (52.84%)	4016 (63.69%)	4478 (70.89%)	4872 (77.17%)	
Female	2977 (47.16%)	2290 (36.31%)	1839 (29.11%)	1441 (22.83%)	
Smoking status					< 0.001
Current smoker	825 (13.07%)	1187 (18.82%)	1532 (24.25%)	1939 (30.71%)	
Ever smoker	504 (7.98%)	500 (7.93%)	542 (8.58%)	502 (7.95%)	
Never smoker	4983 (78.94%)	4619 (73.25%)	4243 (67.17%)	3872 (61.33%)	
Drinking status					< 0.001
Current drinker	462 (7.32%)	515 (8.17%)	576 (9.12%)	673 (10.66%)	
Ever drinker	963 (15.26%)	1055 (16.73%)	1227 (19.42%)	1289 (20.42%)	
Never drinker	4887 (77.42%)	4736 (75.10%)	4514 (71.46%)	4351 (68.92%)	
Family history of diabetes					0.633
No	6161 (97.61%)	6155 (97.61%)	6148 (97.32%)	6164 (97.64%)	
Yes	151 (2.39%)	151 (2.39%)	169 (2.68%)	149 (2.36%)	

Continuous variables were summarized as mean (SD) or medians (quartile interval); categorical variables were displayed as percentage (%)

*FPG* fasting plasma glucose, *DBP* diastolic blood pressure, *BMI* body mass index, *TC* total cholesterol, *TG* triglyceride, *SBP* systolic blood pressure, *TyG* the triglyceride-glucose index, *AST* aspartate aminotransferase, *ALT* alanine aminotransferase, *LDL-C* low-density lipid cholesterol, *BUN* blood urea nitrogen, *Scr* serum creatinine, *HDL-C* high-density lipoprotein cholesterol

**Fig. 2:**
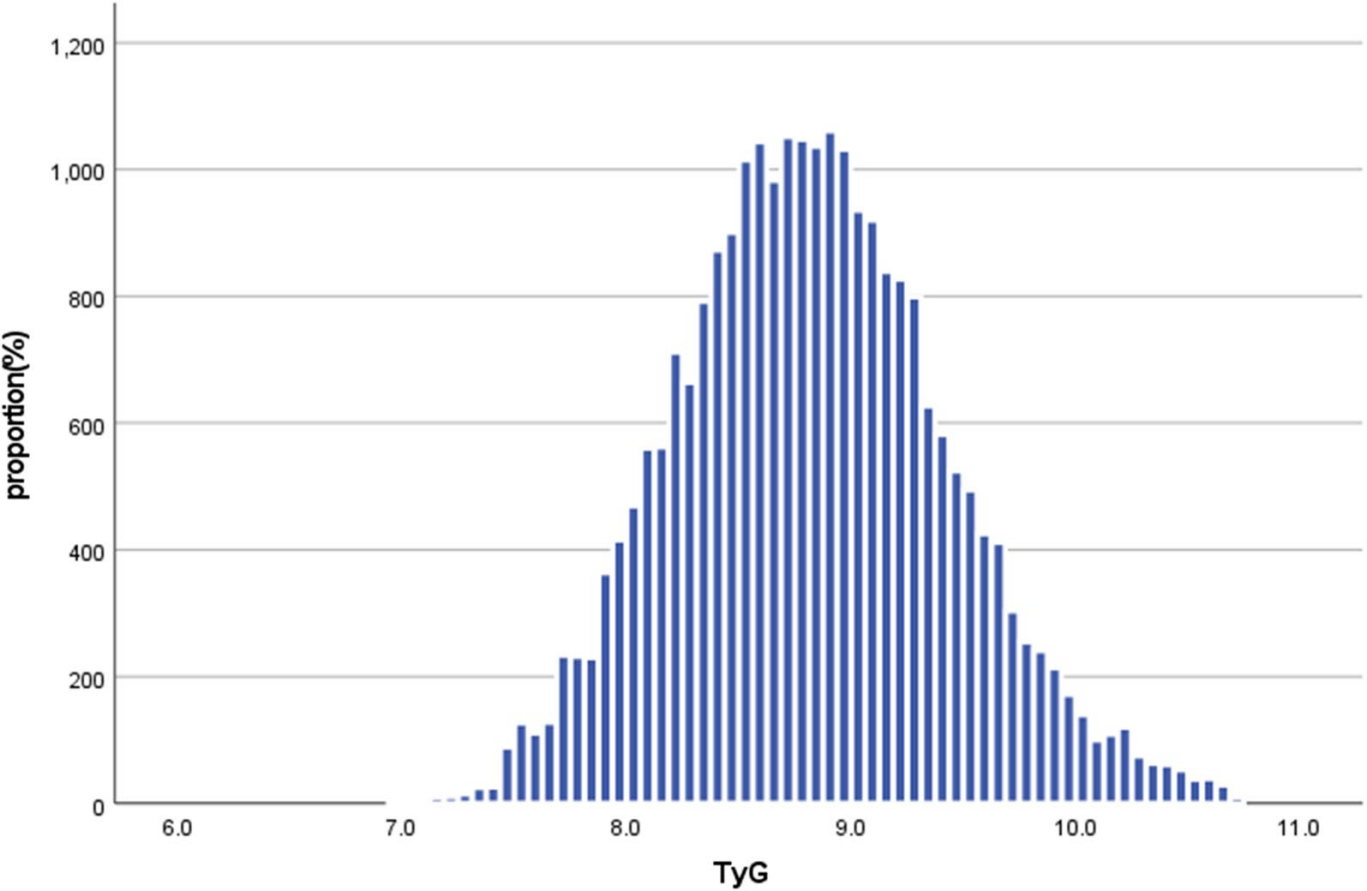
Showed the distribution of TyG levels. It showed a normal distribution within a range from 6.98 to 10.71 (with an average of 8.83). Participants were divided into two groups according to whether they experienced a final diagnosis of normoglycemia

Additional file 1: Fig S1 shows the distribution of TyG for the two groups. The results indicated that the distribution level of TyG was higher in the non-normoglycemia group. On the contrary, the group with normoglycemia had a lower distribution level of the TyG. Figure 2 showed the distribution of TyG levels. It showed a normal distribution within a range from 6.98 to 10.71 (with an average of 8.83).

### The incidence rate of glucose status conversion from pre-diabetes to normoglycemia

Table 2 revealed that 11,499 (45.54%) participants had experienced glucose status conversion from pre-diabetes to normoglycemia during a median follow-up time of 2.96 years. The total cumulative incidence rate of all participants was 15.39 per 100 person-years. Respectively, the cumulative incidence of the four TyG quartile groups were 19.91, 16.24, 14.02, and 11.47 per 100 person-years.

**Table 2:** incidence rate of normoglycemia in paticipants with pre-diabetes

TyG	Participants (n)	Normalglycemia events (n)	Incidence rate (95% CI) (%)	Per 100 person-year
Total	25248	11499	45.54 (44.9–46.2)	15.39
Q1 (< 8.41)	6312	3694	58.52(57.3–59.7)	19.91
Q2 (8.41–8.81)	6306	3011	47.75 (46.5–49.0)	16.24
Q3 (8.81–9.22)	6317	2622	41.51 (40.3–42.7)	14.02
Q4 (> 9.22)	6313	2172	34.41 (33.2–35.6)	11.47
P for trend			< 0.001	

*TyG* triglyceride-glucose index, *CI* confidence interval

**Fig. 3:**
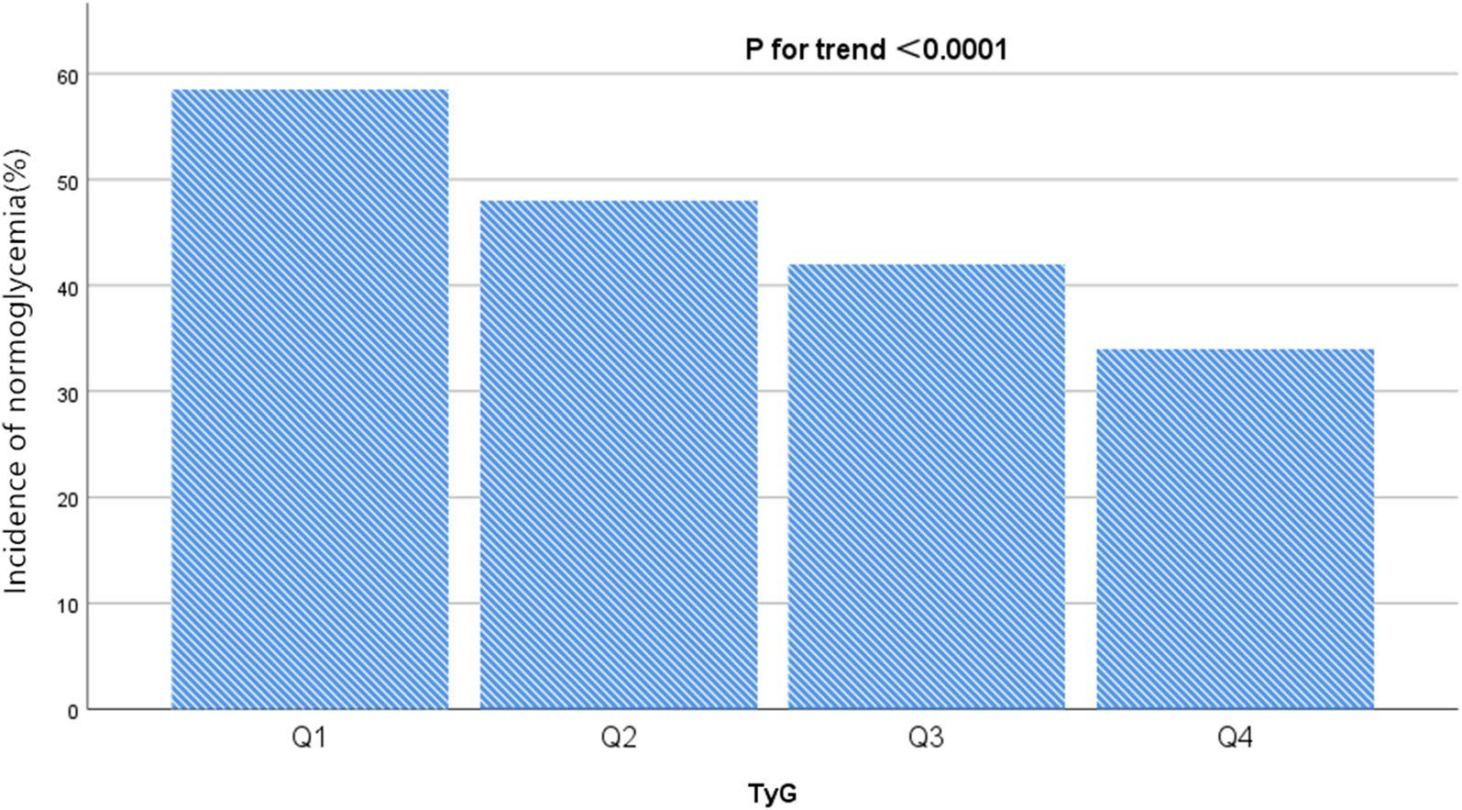
Incidence of events according to the quartiles of TyG. Participants in the high TyG group had a lower blood glucose recovery incidence than the lowest TyG group. Incidence of events were compared by χ^2^ (categorical variables) test. (p < 0.0001 for trend)

The incidence rate (%) of each TyG group was 58.52 (95% CI 57.3–59.7), 47.75 (95% CI 46.5–49.0), 41.51 (95% CI 40.3–42.7), 34.41 (95% CI 33.2–35.6), respectively. Participants within the highest TyG group had lower incidence rates of glucose status conversion compared to the group with the lowest TyG (*p* < 0.0001 for trend) (Fig. 3).

**Fig. 4:**
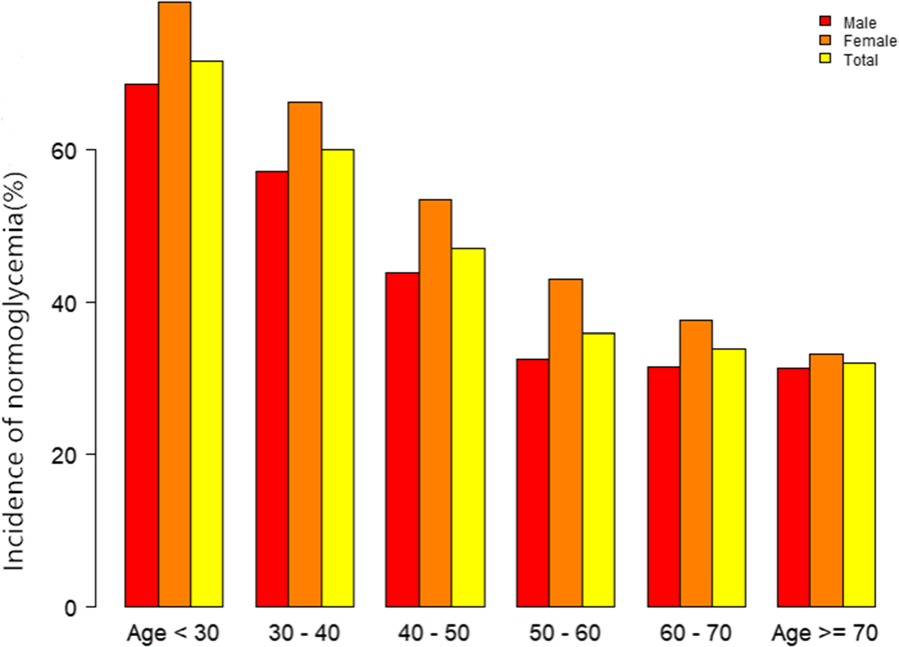
Normoglycemia incidence rate of age stratification by 10 intervals. This figure showed that in age stratification by 10 intervals, female subjects had a higher incidence of normoglycemia incidence rate than male subjects no matter what age group they were in. It also found that the incidence of normoglycemia decreased with age, both in males and females participants

When stratifying participants by 10 age intervals, female subjects had a higher incidence of normoglycemia than male subjects no matter what age group they were in Fig. 4. It also found that the incidence of normoglycemia decreased with age, both in males and females participants.

### The results of univariate analyses using cox proportional-hazards regression model

The univariate analyses showed that incidence of normoglycemia was positively related to female (HR = 1.259, 95% CI 1.212, 1.307), HDL-c (HR = 1.961, 95% CI 1.860, 2.067); and negatively related to Age (HR = 0.977, 95% CI 0.975, 0.978), BMI (HR = 0.937, 95% CI 0.931, 0.942), SBP (HR = 0.990, 95% CI 0.989, 0.991), DBP (HR = 0.985, 95% CI 0.983, 0.987), TC (HR = 0.877, 95% CI 0.860, 0.895), TyG (HR = 0.693, 95% CI 0.672, 0.715), LDL-C (HR = 0.930, 95% CI 0.906, 0.955), ALT (HR = 0.99, 95% CI 0.99, 0.99), AST (HR = 0.99, 95%CI 0.98, 0.99), BUN (HR = 0.957, 95% CI 0.943, 0.972), Scr (HR = 0.997, 95% CI 0.996, 0.998), family history of diabetes (HR = 0.767,95% CI 0.677, 0.868), history of smoking (HR = 0.873, 95% CI 0.838, 0.909) or drinking (HR = 0.858, 95% CI 0.822, 0.894) (Table 3).

**Table 3:** Factors related to blood glucose recovery in patients with prediabetes

Exposure	Statistics	HR(95% CI)	P value
Age (years)	49.273 ± 13.838	0.977 (0.975, 0.978)	< 0.0001
Sex			
Male	16701 (66.148%)	1.0	
Female	8547 (33.852%)	1.259 (1.212, 1.307)	< 0.0001
BMI (kg/m^2^)	24.805 ± 3.365	0.937 (0.931, 0.942)	< 0.0001
SBP (mmHg)	127.205 ± 17.613	0.990 (0.989, 0.991)	< 0.0001
DBP (mmHg)	78.376 ± 11.147	0.985 (0.983, 0.987)	< 0.0001
TC (mmol/L)	4.967 ± 0.943	0.877 (0.860, 0.895)	< 0.0001
TyG	8.827 ± 0.601	0.693 (0.672, 0.715)	< 0.0001
HDL-C (mmol/L)	1.328 ± 0.303	1.961 (1.860, 2.067)	< 0.0001
LDL-C (mmol/L)	2.890 ± 0.718	0.930 (0.906, 0.955)	< 0.0001
ALT (U/L)	28.375 ± 23.445	0.993 (0.992, 0.994)	< 0.0001
AST (U/L)	26.557 ± 12.051	0.986 (0.985, 0.988)	< 0.0001
BUN (mmol/L)	4.989 ± 1.250	0.957 (0.943, 0.972)	< 0.0001
Scr (μmol/L)	72.699 ± 15.984	0.997 (0.996, 0.998)	< 0.0001
Smoking status			
Never smoker	17717 (70.172%)	1.0	
Current or ever smoker	7531 (29.828%)	0.873 (0.838, 0.909)	< 0.00001
Drinking status			
Never drinker	18488 (73.226%)	1.0	
Current or ever drinker	6760 (26.774%)	0.858 (0.822, 0.894)	< 0.00001
Family history of diabetes			
No	24628 (97.544%)	1.0	
Yes	620 (2.456%)	0.767 (0.677, 0.868)	0.00003

Continuous variables were summarized as mean (SD) or medians (quartile interval); categorical variables were displayed as percentage (%)

*FPG* fasting plasma glucose, *BMI* body mass index, *SBP* systolic blood pressure, *DBP* diastolic blood pressure, *TC* total cholesterol, *TG* triglyceride, *TyG* the triglyceride-glucose index, *BUN* blood urea nitrogen, *LDL-c* low-density lipid cholesterol, *Scr* serum creatinine, *HDL-c* high-density lipoprotein cholesterol, *AST* aspartate aminotransferase, *ALT* alanine aminotransferase

**Fig. 5:**
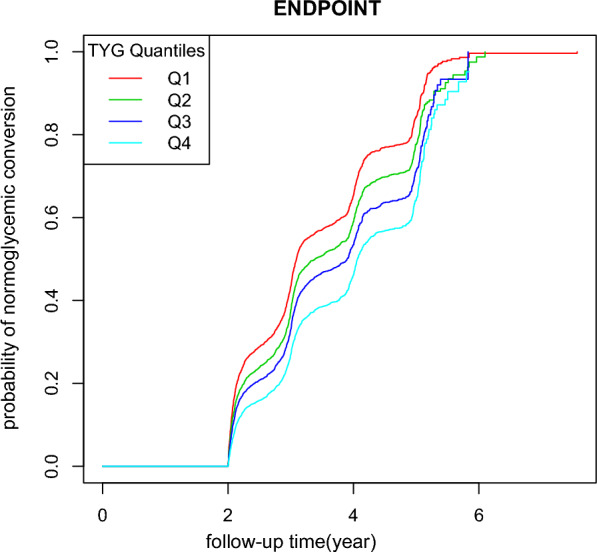
Kaplan–Meier survival curve. The probability of normoglycemic conversion survival differed significantly between the TyG quartiles (log-rank test, p < 0.001).The probability of normoglycemia survival gradually decreased with increasing TyG, suggesting that the group with the highest TyG had the lowest probability of normoglycemic conversion

Kaplan–Meier survival curves for normoglycemia-free survival probability stratified by the TyG group were shown in Fig. 5. There were significant differences in the probability of normoglycemia-free survival between the TyG quartile groups (log-rank test, p < 0.0001).The probability of normoglycemia-free survival gradually decreased with decreasing TyG, indicating that the group with the highest TyG had the lowest rate of normaglycemia converting from prediabetes.

### The results of multivariate analyses using cox proportional-hazards regression model

Three Cox proportional-hazards regression models were built to explore the relationship between TyG and incident normaglycemia (Table 4). In the unadjusted model (Crude model), an increase of 1 unit of TyG was connected with a 30.7% decrease in probability of normaglycemia converting from prediabetes. (HR = 0.693, 95% CI 0.672–0.715, *P* < 0.00001). In Model I, we adjusted for demographic variables (age, sex, BMI, SBP, DBP), each additional unit of TyG decreased by 15.4% in probability of normaglycemia converting from prediabetes. (HR = 0.846, 95% CI 0.817–0.875, *P* < 0.00001). While in fully adjusted model (Model II), unit of TyG decreased by 15.4% in probability of normaglycemia converting from prediabetes (HR = 0.895, 95% CI 0.863–0.928, *P* < 0.00001).

**Table 4:** relations between TyG and the indidence of normalization of blood glucose

Exposure	Crude model	Model I (HR, 95% CI, *P*)	Model II (HR, 95% CI, *P*)	Model III (HR, 95% CI, *P*)
TyG	0.693 (0.672, 0.715) < 0.00001	0.846 (0.817, 0.875) < 0.00001	0.895 (0.863, 0.928) < 0.00001	0.918 (0.885, 0.953) < 0.00001
TyG quartile				
Q1	1.0	1.0	1.0	1.0
Q2	0.818 (0.780, 0.859) < 0.00001	0.973 (0.925, 1.022) 0.27115	0.989 (0.941, 1.040) 0.66878	1.005 (0.955, 1.057) 0.84787
Q3	0.698 (0.664, 0.734) < 0.00001	0.901 (0.854, 0.951) 0.00014	0.964 (0.912, 1.019) 0.19605	0.987 (0.933, 1.044) 0.65351
Q4	0.557 (0.528, 0.587) < 0.00001	0.755 (0.712, 0.800) < 0.00001	0.829 (0.780, 0.882) < 0.00001	0.864 (0.812, 0.920) < 0.00001
*P* for trend	< 0.00001	< 0.00001	< 0.00001	0.00002

Crude model: we did not adjust other covariates

Model I: we adjusted age, sex, BMI, SBP, DBP

Model II: we adjusted age, sex, BMI, SBP, DBP, ALT, AST, BUN, LDL-C, HDL-c, Scr, family history of diabetes, drinking status, and smoking status

Model III: we adjusted age (smooth), sex, BMI, SBP(smooth), DBP(smooth), ALT, AST, BUN, LDL-C(smooth), HDL-c(smooth), Scr(smooth),family history of diabetes, drinking status, and smoking status

*HR* Hazard ratios, *CI* confidence

In addition, when we set the lowest quartile as a reference, the highest quintile of TyG was distinctly associated with decreased possibility of normaglycemia (Crude model, Q5: OR = 0.557, 95% CI 0.528–0.587, *P* < 0.001) (Model I, Q5: OR = 0.755, 95% CI 0.712–0.800, *P* < 0.001) (Model II, Q5: OR = 0.829, 95% CI 0.780–0.882, *P* < 0.001). The results also showed that the trends consistent with the result when TyG categorical values was continuous variables. (Table 4).

### Sensitivity analysis

We used a GAM to insert the continuity covariate into the equation as a curve. The result remained consistent with the fully adjusted model (Table 4) (Model III, HR = 0.918, 95% CI 0.885–0.953, *P* < 0.00001). Besides, the authors excluded participants with BMI ≥ 25 mmol/L for sensitivity analyses, TyG was also negatively associated with normoglycemia converting possibility after adjusting the confounding factors (Model I, HR = 0.918, 95% CI 0.876–0.961, *P* = 0.00030) (Table 5).

**Table 5:** Relationship between TyG and normoglycemic conversion in different sensitivity analyses

Exposure	Model I (HR, 95% CI, *P*)	Model II (HR, 95% CI, *P*)	Model III (HR, 95% CI, *P*)	Model IV (HR, 95% CI, *P*)
TyG	0.918 (0.876, 0.961) 0.00030	0.859 (0.802, 0.920) 0.00001	0.897 (0.839, 0.959) 0.00138	0.917 (0.883, 0.951) < 0.00001
TyG quartile				
Q1	1.0	1.0	1.0	1.0
Q2	0.980 (0.923, 1.040) 0.50080	1.025 (0.924, 1.137) 0.63880	0.936 (0.843, 1.040) 0.21861	1.010 (0.959, 1.063) 0.71689
Q3	0.955 (0.890, 1.024) 0.19730	0.972 (0.871, 1.084) 0.60592	0.912 (0.817, 1.017) 0.09665	0.992 (0.938, 1.050) 0.79377
Q4	0.848 (0.781, 0.921) 0.00010	0.782 (0.695, 0.879) 0.00004	0.843 (0.752, 0.945) 0.00344	0.863 (0.810, 0.919) < 0.00001
*P* for trend	0.00039	0.00003	0.00369	0.00002

Model I was a sensitivity analysis performed after excluding participants with BMI ≥ 25 kg/m2. we adjusted age, sex, SBP, DBP, ALT, AST, BUN, LDL-C, HDL-c, Scr,family history of diabetes, drinking status, and smoking status

Model II was a sensitivity analysis performed on participants who had never consumed alcohol. we adjusted age, sex, BMI, SBP, DBP, ALT, AST, BUN, LDL-C, HDL-c, Scr, family history of diabetes, and smoking status

Model III was a sensitivity analysis performed on participants who had never smoked. we adjusted age, sex, BMI, SBP, DBP, ALT, AST, BUN, LDL-C, HDL-c, Scr, family history of diabetes, and drinking status

Model IV was a sensitivity analysis performed on participants without a family history of diabetes. we adjusted age, sex, BMI, SBP, DBP, ALT, AST, BUN, LDL-C, HDL-c, Scr, smoking status, and drinking status

*HR* Hazard ratios, *CI* confidence

Participants who had never consumed alcohol were also excluded for sensitivity analyses. The results remained consistent after adjusting the confounding factors (Model II, HR = 0.859, 95% CI 0.802, 0.920, *P* = 0.00001) (Table 5).

Model III on Table 5 was a sensitivity analysis performed on participants who had never smoked. We still got similar results (Model III, HR = 0.897, 95% CI 0.839–0.959, *P* = 0.00138).

participants without a family history of diabetes were also performed for sensitivity analyses, TyG also had negative association with normoglycemia converting from prediabetes after adjusting the confounding factors (Model IV, HR = 0.917, 95% CI 0.883–0.951, *P* < 0.00001) (Table 5).

The results obtained from all of the sensitivity analyses indicated the well-robustness of the relationship between TyG and normoglycemia convert.

### The non-linearity addressed by Cox proportional hazards regression model with cubic spline functions

**Fig. 6:**
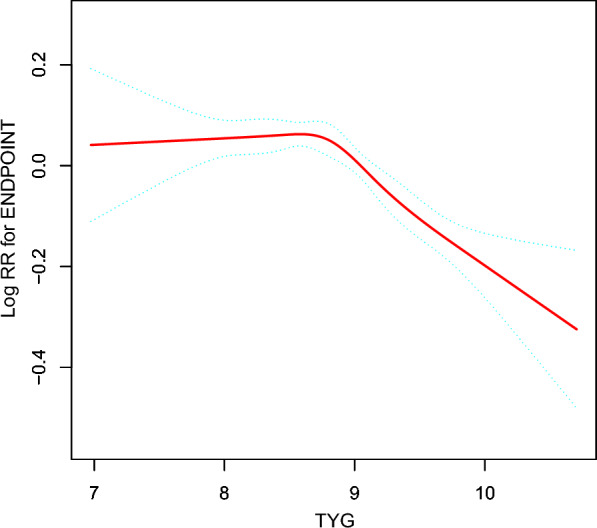
The non-linear relationship between eGFR and the normoglycemic conversion from prediabetes. We used a Cox proportional hazards regression model with cubic spline functions to evaluate the relationship between TyG and recovery of blood glucose incidence. The result showed that the relationship between TyG and recovery of blood glucose incidence was non-linear, with the inflection point of TyG index being 8.88

A non-linear relationship was detected between TyG and normoglycemia convert by the Cox proportional hazards regression model with cubic spline functions analyses (Fig. 6). To determine the best fit model using a log-likelihood ratio test, it turns out that the P for the log-likelihood ratio test was < 0.001. By recursive algorithm, we got the inflection point 8.88 (Table 6).

**Table 6:** the result of two-piecewise regression linear model

Indidence of normalization of blood glocose	HR, 95% CI	*P*
Fitting model by standard Cox regression	0.90 (0.86, 0.93)	< 0.0001
Fitting model by two-piecewise Cox regression		
Inflection points of TyG	8.88	
< 8.88	0.99 (0.93, 1.05) 0.7895	0.7456
> 8.88	0.79 (0.74, 0.85)	< 0.0001
*P* for log-likelihood ratio test	0.80 (0.72, 0.89)	< 0.0001

We adjusted age, sex, BMI, SBP, DBP, ALT, AST, BUN, LDL-C, HDL-c, Scr, family history of diabetes, drinking status, and smoking status

On the right side of the inflection point by two piecewise Cox proportional-hazards regression model, the HR and 95% CI were 0.79 (0.74, 0.85). That indicates when TyG value is greater than 8.88, the probability of normoglycemia convert events become smaller as TyG value increases.

While on the left side of the inflection point by two piecewise Cox proportional-hazards regression model, the relationship between TyG and normoglycemia convert was not statistically significant (HR = 0.99, 95% CI 0.93–1.05, *P* > 0.05). (Table 6).

### The results of subgroup analyses

**Fig. 7:**
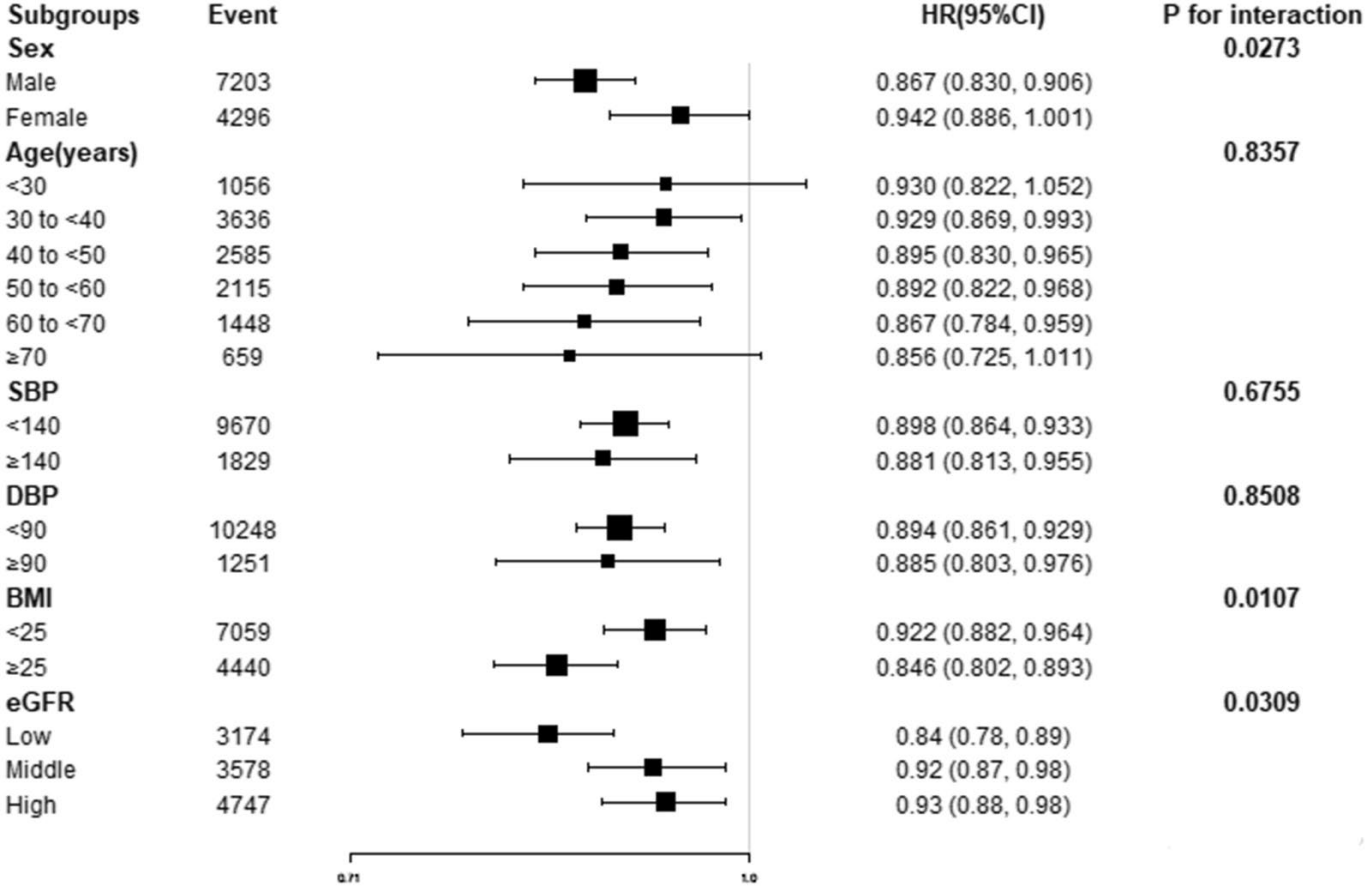
Effect size of TyG on normoglycemia in prespecified and exploratory subgroups. The subgroup analyses were conducted using a stratified Cox proportional-hazards regression model across various subgroups. The model above is adjusted for age, sex, BMI, SBP, DBP, ALT, AST, BUN, LDL-C, HDL-c, Scr, family history of diabetes, drinking status, and smoking status, but not adjusted for stratification variables

Subgroup analyses showed that there were no significant interaction between TyG and incident normoglycemia convert according to strata of age, SBP, DBP (Fig. 7).

The results showed that Sex and BMI could modify the relationship between TyG and incident normoglycemia convert (All P for interaction < 0.05). And a stronger association was observed in males (OR = 0.867, 95% CI 0.830–0.906) and the participants with BMI ≥ 25 kg/m^2^ (OR = 0.846, 95% CI 0.802–0.893), and with lower eGFR (OR = 0.84, 95% CI 0.78–0.89). In contrast, weaker association was observed in females (HR = 0.942, 95% CI 0.886–1.001), participants with BMI < 25 kg/m^2^ (OR = 0.922, 95% CI 0.882–0.964), and with higner eGFR (OR = 0.93, 95% CI 0.88–0.98) (Fig. 7).

## Discussion

The purpose of this study was to investigate the relationship between TyG and blood glucose normalization in prediabetic patients. Our findings indicate a negative correlation between TyG levels and glucose normalization in prediabetic patients. Higher TyG levels were associated with a lower likelihood of glucose-normalizatio during follow-up, whereas lower TyG levels were associated with a greater tendency for glucose-normalization. Furthermore, we observed a non-linear relationship between TyG and glucose-normalization, with an inflection point at a value of 8.88. When TyG was greater than 8.88, the incidence of glucose-normalization decreased as TyG values increased. Additionally, subgroup analysis revealed that BMI and gender acted as influencing modifiers. Specifically, in male patients and patients with a BMI ≥ 25, TyG exhibited a stronger negative correlation with blood glucose normalization. In other words, lower TyG levels were associated with a higher likelihood of blood glucose normalization in those subgroup.

Prediabetes is a high risk factor for progression to diabetes. 5–10% of people with prediabetes developes diabetes each year. Prediabetes is closely related to eyes, kidneys and cardiovascular diseases. Therefore, it is important to pay attention to prediabetes and monitor risk indicators associated with prediabetes. Previous literatures have shown that prediabetes can be reversed to normoglycemia. In an Indian study, 32 (26.67%) of 120 patients with prediabetes had normalized blood glucose after 32 months of follow-up [26]. In a Swedish study of 918 elderly patients (age 60 years or older) with prediabetes, 204 (22%) reverted to normoglycemia, 119 (13%) developed diabetes, and 215 (23%) died after 12 years of follow-up. The incidence of blood glucose normalization, progression to diabetes, and death was higher during the first 6 years of follow-up than during the second 6 years of follow-up [27].In addition, it has reported that the normalization rate of blood glucose in middle-aged people is 23–30% [28]. In our study, after a median follow-up time of 2.96 years, 11,499 (45.54%) prediabetic patients were finally diagnosed with normalized blood glucose, which is higher than the conversion rate of 23–30% reported in the literature above. The observed variation in the relationship between TyG and blood glucose normalization in prediabetic patients might be attributed to several factors, including differences in ethnic groups, age groups, definitions of prediabetes, as well as different lifestyle and medications. Lifestyle interventions and medications have been reported to reduce the risk of progression to diabetes and increase the incidence of blood glucose normalization in patients with prediabetes. Therefore, in a certain range, the incidence of conversion of blood glucose normalization in prediabetes patients is affected by different lifestyle and drug therapies. However, further research is needed to confirm the main contributing factors in this regard.

In a cohort study based on a Chinese population, TyG was strongly associated with an increased risk of diabetes, with a HR of 3.34 (95% CI 3.11–3.60) for diabetes [29]. Another Korean study found that TyG could be used as a predictor of type 2 diabetes in non-obese adults. This study divided the population into four groups according to the TyG4 quantiles and when compared with group Q1 (HR = 1.0), Q2 (HR = 1.63, 95% CI 1.18 2.24), Q3 (HR = 2.3, 95% CI 1.68 3.14) and Q4 group (HR = 3.67, 95% CI 2.71 4.98) had higher rates of progressing to diabetes [30]. Studies have also found that increased TyG level is closely related to the risk of prediabetes, and the risk of progressing to prediabetes increased by 38% for every 1 SD increase of TyG (HR = 1.38, 95% CI 1.28–1.48) [31]. It was also found that TyG-BMI was an independent predictor of new-onset diabetes (HR 1.50 per SD increase, 95% CI 1.40–1.60, P-trend < 0.00001) [32]. In the Indonesia study, TyG index appears to be a promising marker for glucose status conversion among reproductive-aged women [19]. We have the similar results with the previous literatures. In our study, it was found that TyG was negatively correlated with the normalization of blood glucose, and there was a nonlinear correlation between TyG and conversion to normoglycemia in prediabetes people, with an infection point of 8.88. At the same time, sensitivity analysis was also conducted in our study. It was found that TyG was negatively correlated with blood glucose normalization regardless of family history of diabetes, smoking history, alcohol consuming history and obesity (BMI ≥ 25). The results of sensitivity analysis indicated the robustness between TyG and conversion to normoglycemia in prediabetes people. It is important to highlight that this study primarily investigated the impact of TyG on the reversal to normoglycemia in individuals with prediabetes. The findings of this study have clinical significance as they provide insights into early interventions for prediabetic individuals, facilitating their transition to normoglycemia and potentially preventing rapid progression to diabetes.

However, neither literature studying the effects on TyG predicting the conversion from prediabetes to normal blood glucose, nor the literature reporting the relationship between TyG and conversion from prediabetes to noroglycemia in Chinese people. The mechanism by which TyG predicts normoglycemia is uncertain, but it is known that IR plays an important role in the conversion from prediabetes to normal blood glucose. We speculated that the relationship between TyG and blood glucose reversal was mediated by IR. First, TyG has a strong ability to predict IR. In Mexican population, compared with hyperinsulinemic-euglycemic clamp, which is the gold standard of IR measurement, the sensitivity of TyG to predict IR is as high as 96.5% [21]. IR is the pathophysiological basis of abnormal blood glucose, which can explain why TyG has a strong predictive ability for prediabetes. TyG is a compound index of triglyceride and fasting blood glucose. Triglyceride can be degraded into glycerol and fatty acids, while the increased free fatty acids can be transferred from adipose tissue to non-adipose tissue, thus leading to insulin resistance. Hypertriglyceridemia leads to transport of high free fatty acids to the liver leading to high glucose output in the liver, that is, free fatty acids can increase gluconeogenesis in the liver [33], and high levels of triglycerides in the liver and muscle can interfere with glucose metabolism in each target organ. In addition, glucotoxicity and lipotoxicity can lead to increased production of reactive oxygen species, resulting in oxidative stress and β cell dysfunction. A reduction in the antioxidant defense of pancreatic beta cells can ultimately lead to IR. Long-term exposure to high concentrations of free fatty acids and long-term exposure to total cholesterol can lead to impaired beta cell function and reduced glucose-induced insulin secretion [34–36].

Subgroup analysis suggested that TyG was more strongly associated with glucose normalization in male patients (OR = 0.867, 95% CI 0.830–0.906). In female patients, the relationship was weaker (OR = 0.942, 95% CI 0.886, 1.001). Previous literature has reported that compared with women, men have a higher risk of developing type 2 diabetes, which is speculated to be due to higher insulin resistance in men [37]. That means women need to gain more weight and fat tissue to develop insulin resistance and then progress to type 2 diabetes. In addition, the distribution and quality of fat varies by gender, and in general, men have more visceral fat and liver fat than women. In men, ectopic fat accumulates in different organs including the liver, which determines the increase of liver enzymes and triglycerides, causing the organ to develop insulin resistance [38]. Toxic lipid derivatives (ceramides or diglycerides) or other molecules are involved in this process and regulate insulin pathway. Besides, HDL-C levels are generally lower in men than in women (accompanied by higher fasting glucose levels) [38]. Obesity and insulin resistance are important mediators in the development of type 2 diabetes in male, and also affect the outcome of blood glucose normalization. TyG is a complex index of blood glucose and lipid, and multiple studies have confirmed that TyG is closely related to IR, and TyG can reflect the function of IR and pancreatic β cells. Men have higher insulin resistance, and insulin resistance is highly correlated with TyG, so the reversion status of abnormal glycemia blood glucose can be better reflected by TyG in male rather than female.

By subgroup analysis, we also found that when BMI ≥ 25, TyG had a stronger relationship with blood glucose normalization (OR = 0.846, 95% CI 0.802, 0.893). When BMI < 25, the relationship was weaker (OR = 0.922, 95% CI 0.882, 0.964). BMI is an indicator of obesity. Higher visceral fat might enter and accumulate in the liver via the portal system, which increases liver TG levels, reduces insulin reabsorption, causes metabolic disturbances, and increases the risk of IR, thus finally leads to obesity. Excess visceral fat, in turn, promotes inflammation in the body by increasing certain cytokines. Meanwhile, macrophages in visceral adipose tissue secrete cytokines, including IL-6 and TNF-α, which can stimulate chronic inflammatory response, followed by the production of IR [39]. Whether prediabetes could reverse to normoglycemia is closely related to IR degree, while BMI is closely related to obesity, chronic inflammatory reaction and IR. Therefore, TyG can better reflect the outcome of reversion of normoglycemia in patients with higher BMI.

More importantly, our study is the first study to identify a non-linear relationship between TyG and reversion to normoglycemia in patients with prediabetes. After controlling a series of confounding factors, we found that the infection point value of TyG in predicting blood glucose normalization was 8.88. When TyG > 8.88, the possibility of reversion to normoglycemia was reduced by 21% for each additional unit of TyG. When TyG < 8.88, the increase or decrease of TyG had no statistical difference in the occurrence of reversion to normoglycemia with prediabetes. Compared with those with TyG < 8.88, those with TyG > 8.88 were older, had a greater proportion of BMI, SBP, DBP, LDL-c, ALT, AST, BUN, Scr, smoking history, drinking history, and had a lower proportion of HDL-c.

In our study, decreased TyG is significantly correlated with higher nomoglycemic conversion rate with prediabetes in a certain range. But when TyG < 8.88, the risk factors had no statistical influence on the outcome of reversion. Since TyG is a compound index of triglyceride and fasting blood glucose, we speculate that decreased FBG and/or decreased triglyceride might also increase the risk of IR and thus counteract the benefits. In fact, IR has been described in conditions not related to obesity or diabetes, such as starvation, malnutrition, chronic renal failure, where insulin levels may be normal or low. IR is also seen in conditions where body fat is often low, including congenital lipodystrophies, anorexia nervosa, and chronic stress conditions [40].

A previous study showed that in 35 healthy, sedentary, lean, collegeage subjects, 2.3% were IR by HOMA-IR > 2.1, 5.7% by ISI Matsuda < 5.9, and 22.9% had > one criteria for metabolic syndrome (MetS); 28.6% had some negative metabolic biomarker. Low body fat and hypoglycemia have been found to counteract the metabolic action of insulin and are considered to be a factor contributing to the development of IR and associated metabolic abnormalities [41]. In short, when TyG < 8.88, low TyG might increase the risk of IR, hence we could not discover significantly relationship between TyG and nomoglycemia conversion among prediabetes people. The nonlinear relationship between TyG and blood glucose normalization in patients with prediabetes had great clinical significance. It promotes clinical consultation and optimization of decision-making in the prevention of prediabetes/diabetes, and its inflection point provides clinicians with the prognosis of patients with prediabetes. From the perspective of treatment, controlling blood glucose and triglyceride levels through medications or lifestyle interventions to maintain TyG around 8.88 may effectively reduce the risk of patients progressing from prediabetes to diabetes.

The advantages of our study are as follows: (1) Our study is a large sample size study. (2) For the first time, our study described the relationship between TyG and the reversion to normoglycemia from prediabetes in Chinese people, and found the inflection point of TyG (TyG inflection point 8.88) through non-linear correlation statistic analysis, which provided prognostic prediction for screening prediabetes patients as well as provided strong data support for reducing the occurrence of prediabetes/diabetes. (3) Sensitivity analysis was used to evaluate the reliability and stability of our results. (4) We used subgroup analysis and found a stronger association between TyG and glycemic normalization outcomes in specific populations (males, BMI ≥ 25).

However, there are some shortcomings in our study: (1) Prediabetes includes two characteristics of impaired fasting glucose (IFG) and/or impaired glucose tolerance, but only fasting glucose data can be obtained in our database. In this study, we defined prediabetes by impaired fasting glucose, so we failed to identify those with impaired glucose tolerance, which may underestimate the prediabetes population at baseline. Despite its shortcomings, fasting glucose screening for prediabetes remains the most commonly used method because it is safe, convenient, inexpensive, and quick. (2) As all the other observational studies, although known potential confounders such as BMI, SBP, and age were controlled, there were still uncontrolled or unmeasured confounders. (3) The patient's medication regimen (hypoglycemic drugs, lipid-lowering drugs, uric-lowering drugs, antihypertensive drugs, etc. was not included in the follow-up, and the lifestyle which includes diet and exercise would also make influence on the outcome. In short, we still could not judge the effects on the reversion of normoglycemia with drugs and lifestyle. (4) There are some limiting conditions applying TyG to practice in all populations due to the Chinese population trending to have lower BMI, and in our study, obese people were excluded, which is an essential factor in developing insulin resistance and lipid disbalance. So the trend between TyG and glucose normalization or the threshold value of TyG might be changed when more obese people are included in the study. (5) Further studies are needed to improve the diagnosis and prediction of diabetes development in other populations in other countries where it is easy and chip the quantification of glucose and triglycerides instead of an insulin resistance test.

## Conclusion

The incidenct rate of prediabetes progressing to T2DM is high. T2DM is associated with multiple complications, increasing the risk of cardiovascular events and all-cause mortality. However, the abnormal blood glucose in prediabetic people may be reversed, so early identification and intervention of high-risk prediabetic people may delay or even reverse the abnormal glycemia status. Our study showed that TyG index was a good and simple index to predict the blood glucose outcome of patients with prediabetes, so clinicians could consider widely applying TyG index to the clinical expected reversal of blood glucose normalization. When TyG > 8.88, patients with prediabetes are less likely to get reversion to normoglycemia. Therefore, instead of maintaining pre-diabetes or progressing to diabetes, people with prediabetes might benefit from limiting the TyG value to around 8.88.

### Supplementary Information

**Additional file 1: Figure S1.** Distribution of TyG for non-normoglycemic conversion group and normoglycemic conversion group with prediabetes.（0,non-normoglycemia conversion group; 1,normoglycemia conversion group）.

## Data Availability

Data can be downloaded from the ‘DATADRYAD’ database (www.Datadryad.org). The original contributions presented in the study are included in the article/Additional file, further inquiries can be directed to the corresponding author.

## Abbreviations

FPG: Fasting plasma glucose
BMI: Body mass index
SBP: Systolic blood pressure
DBP: Diastolic blood pressure
TC: Total cholesterol
TG: Triglyceride
TyG: The triglyceride-glucose index
BUN: Blood urea nitrogen
LDL-c: Low-density lipid cholesterol
Scr: Serum creatinine
HDL-c: High-density lipoprotein cholesterol
AST: Aspartate aminotransferase
ALT: Alanine aminotransferase
IFG: Impaired fasting glucose
EHC: The euglycemic-hyperinsulinemic clamp
T2DM: Type 2 diabetes mellitus
HbA1c: Hemoglobin A1c
CI: Confidence interval
HR: Hazard ratio
GAM: Generalized additive model
WHO: World Health Organization
ADA: The American Diabetes Association
IFG: Impaired fasting glucose
FPG: Fasting plasma glucose
IR: Insulin resistance
SD: Standard deviation
MAR: Missing-at-random
