# Supplementary material for: Predictive performance of triglyceride glucose index (TyG index) to identify glucose status conversion: a 5-year longitudinal cohort study in Chinese pre-diabetes people
Source: J Transl Med. 2023 Sep 15;21:624. doi: 10.1186/s12967-023-04402-1 (PMC10503019; doi:10.1186/s12967-023-04402-1)
Supplement: Supplementary file 1 — Additional file 1: Figure S1. Distribution of TyG for non-normoglycemic conversion group and normoglycemic conversion group with prediabetes.（0,non-normoglycemia conversion group; 1,normoglycemia conversion group）. [file 12967_2023_4402_MOESM1_ESM.docx]

**Predictive performance of triglyceride glucose index (TyG index) to identify glucose status Conversion: A 5-year longitudinal cohort study in Chinese** **pre-diabetes people.**

**Xiaojie Chen^1,2,3,4,5^ Danfeng Liu^2,3,6^ Weiting He^1,2,3^ Haofei Hu^1,2,3,4,5^ Wenjian Wang^2,3^**

^1^Southern Medical University, Guangzhou, China

^2^Department of Nephrology, Guangdong Provincial People's Hospital, Guangzhou, China

^3^Guangdong Academy of Medical Sciences, Guangzhou, China

^4^Department of Nephrology, the First Affiliated Hospital of Shenzhen University, Shenzhen, China

^5^Department of Nephrology, Shenzhen Second People’s Hospital, Shenzhen, China

^6^South China University of Technology, Guangzhou, China

**Correspondence:**

**Wenjian Wang M. D., Ph. D.**

Division of Nephrology

Guangdong Provincial People’s Hospital

Guangdong Academy of Medical Sciences

106 Zhongshan Er Road

Main Building, Room 1436

Guangzhou, Guangdong, 510080, China

Tel: +86 (20)83827812-61421

E-mail: [wwjph@126.com](mailto:wwjph@126.com)

**Haofei HU** **M. D.**

Department of Nephrology,

Shenzhen Second People’s Hospital,

No.3002 Sungang Road, Futian District,

Shenzhen 518000,

Guangdong Province,

China

Tel:+86-755-83366388

E-mail: [huhaofei0319@126.com](mailto:huhaofei0319@126.com)

**
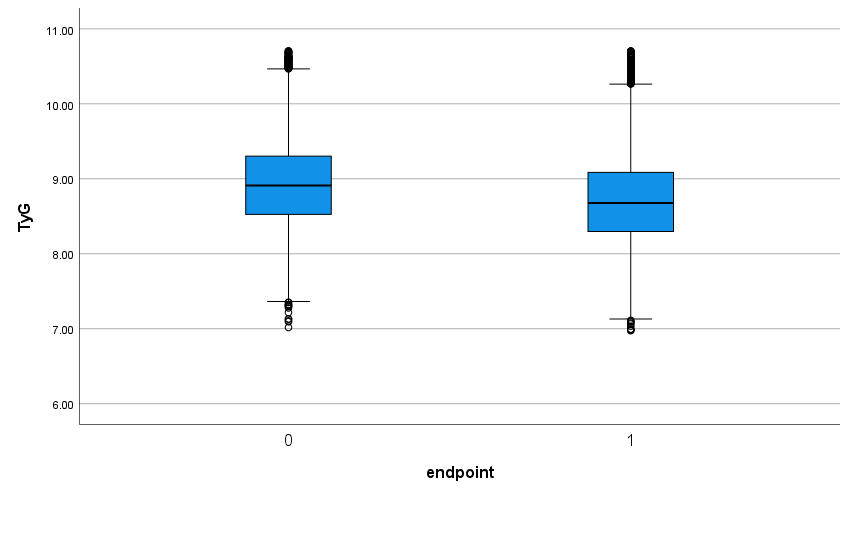
**

**Figure S1** Distribution of TyG for non-normoglycemic conversion group and normoglycemic conversion group with prediabetes.（0,non-normoglycemia conversion group; 1,normoglycemia conversion group）
